# Taking stock of population-level interventions targeting risk factors for hypertension and diabetes in Rwanda and South Africa: methodological reflections and lessons learnt from conducting a multi-component situational analysis

**DOI:** 10.1186/s12889-023-16537-3

**Published:** 2023-08-25

**Authors:** Uwimana Nicol J, Nganabashaka JP, Tumusiime KD, Young T, Rehfuess E, Burns J

**Affiliations:** 1 Division of Epidemiology and Biostatistics, Department of Global Health, Stellenbosch University, Cape Town, South Africa; 2 College of Medicine and Health Sciences, University of Rwanda, Kigali, Rwanda; 3 Institute for Medical Information Processing, Biometry and Epidemiology (IBE), Chair of Public Health and Health Services Research, LMU Munich, Munich, Germany; 4 Pettenkofer School of Public Health, Munich, Germany

**Keywords:** Situational analysis, Population level interventions, Diabetes, Hypertension, Non-communicable diseases, Methodology, Multi-component, Challenges, Lessons learnt, Rwanda and South Africa

## Abstract

**Background:**

Hypertension and diabetes are on the rise both in Rwanda and South Africa. The responsibility for NCD risk factors cut across different sectors, which makes it complex to effectively manage. Policy-relevant intervention research is thus critical for addressing the NCD challenge. We conducted a situational analysis in both countries to identify and describe current population-level interventions targeting risk factors for diabetes and hypertension. This paper presents this methodology and shares challenges encountered, and lessons learnt in applying the methodology.

**Methods:**

We describe a multi-component methodology for conducting a situational analysis, which included a desk review, stakeholder mapping, survey, key informant interviews, and a consultative workshop. This methodology was applied in a standardized manner in two African countries. Following the analysis, the authors held iterative team consultations to reflect on challenges and lessons learnt during this process.

**Results:**

Key challenges and lessons learnt relate to i) stakeholder recruitment, engagement and retainment; ii) utilization and triangulation of multiple sources of data; and iii) evolving circumstances, particularly related to the Covid-19 pandemic. It proved challenging to recruit stakeholders outside the health sector and in the private sector, as they often do not consider themselves as making or influencing policies and thus were reluctant to engage. The difficulties with responsiveness were often overcome through face-to-face visits, an opportunity to explain the relevance of their participation. With regards to health sector stakeholders and all other stakeholders, continued engagement over prolonged periods of time also turned out to be challenging. Covid-19 restrictions were preserved to be an impediment throughout the conduct of the situational analysis, specifically in South Africa. The use of multi-stage mixed methods was found to be appropriate for addressing the study objectives, as each step yielded unique data, concepts, and perspectives that complemented the other data.

**Conclusion:**

Conducting a situational analysis is crucial for understanding the current state of interventions and identifying opportunities for new interventions. The multi-component methodology used in two African countries was found to be feasible, appropriate, and informative. Others planning to conduct situational analysis may follow, adapt and improve upon our approach, reacting to the challenges encountered.

## Background

Non-Communicable Diseases (NCDs) represent a serious public health burden globally with at least 41 million annual deaths, representing 71% of all global deaths [1]. The World Health Organization (WHO) ranks Cardiovascular Diseases (CVDs) as the leading cause of death globally, with an estimated 17.9 million deaths from CVDs in 2019, representing 32% of all global deaths [2]. Hypertension and diabetes, as important risk factors for CVDs, are major contributors to the global NCD burden [3]. Globally, 1.28 billion and 451 million individuals live with hypertension and diabetes, respectively [4, 5]. The global burden from NCDs, however, is not distributed equally, with 77% of all global NCD deaths occurring in Low and Middle-Income Countries (LMICs) [6], including Rwanda, South Africa (SA) and other countries of sub-Saharan Africa (SSA). According to the most recent estimates from the WHO STEPwise approach to NCD risk factor surveillance (STEPS survey), the prevalence of hypertension in adults in Rwanda is 15% and the prevalence of diabetes among 15–64 year-olds in rural and urban areas was estimated at 8% and 10%, respectively [7]. In SA, the South Africa Demographic and Health Survey (SADHS)indicated that the prevalence of hypertension .has nearly doubled since 1998, from 25% to 46% among women and from 23% to 44% among men, while 13% of women and 8% of men age 15 and older have an adjusted HbA1c level of 6.5% or above, indicating that they are diabetic [8].

The aspects highlighted above underline the need for effective interventions to address key risk factors and thus to reduce the burden of hypertension and diabetes, and subsequently CVDs, especially in SSA countries such a Rwanda and SA. Indeed, the World Health Assembly called for a 25% reduction in NCD deaths by 2025, including from CVDs and diabetes [9]. It is clear that population-level interventions addressing modifiable risk factors, both focusing on changes to behavior and lifestyle as well as modifying the environment, will be critical in reducing the burden from NCDs [10, 11]. These population-level interventions cover increasing tobacco taxes, restricting alcohol advertising, reformulating food products with less salt, sugar, and fat, and treating hypertension and diabetes, among others [12]. These types of interventions concern different sectors beyond health including agriculture, sports, infrastructure, commerce and industries and many more [13]. To ensure that appropriate and effective population-level interventions are in place, it is imperative for countries to conduct different types of policy-relevant intervention research. Additionally, the multi-sectoral nature of such interventions necessitates the involvement of all relevant stakeholders in understanding existing and future interventions. Taken together, this underscores the importance of rigorous methodologies and the engagement of stakeholders to identify and understand the existing interventions targeting different NCDs risk factors, as well as how these interventions could be improved. The method of situational analysis represents an important part of this complex research infrastructure [14].

Situational analysis in the context of health interventions is used to identify and understand existing interventions, as well as the multiple interacting factors, including context, linked to these and potential additional interventions [15, 16]. Several situational analyses have been conducted in the SSA context, and have assessed different health policies and interventions , among others, in the area of tobacco control [17], child and adolescent mental health services [18], prostate cancer screening [19] and drug-resistant tuberculosis [20]. While such examples do exist, we argue that the methodology is still underutilized and that there is a lack of detailed descriptions of real-world applications [21].

As part of the Collaboration for Evidence-based Healthcare and Public Health in Africa (CEBHA+), funded by the German Federal Ministry of Education and Research (BMBF), we conducted a multi-component situational analysis in Rwanda and SA in 2019-2020 to identify and describe existing population-level interventions targeting risk factors for diabetes and hypertension. We also aimed to identify gaps and opportunities to effectively advance population-level interventions in both countries. The complete findings of the situational analysis are described in separate papers [22, 23].

In this paper, we describe the methodology as applied in the two countries, as well as reflect upon and describe the challenges encountered and lessons learnt during the process. In view of the situational analysis representing a valuable early step in policy-oriented efforts to identify, select, implement or evaluate the interventions, we suspect that many of the challenges we encountered are common and would be useful to others planning to undertake a situational analysis.

### Description of situational analysis methodology

**Fig. 1:**
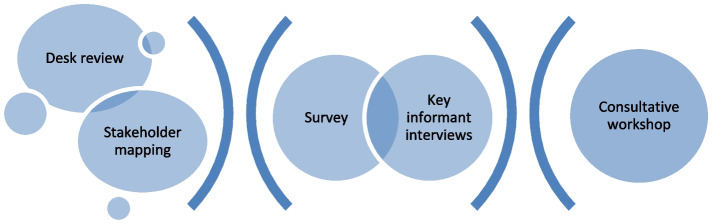
Overview of methods for situational analysis

We conducted a mixed-method situational analysis, incorporating both quantitative and qualitative components. These methods, summarized in Fig. 1, comprised: a desk review, stakeholder mapping, a survey, key informants’ interviews (KIIs) and consultative workshops. Each of these specific methodological components is described in further detail in the following sections.

We developed a common protocol at the outset of the project to clarify the objectives and ensure the application of a similar methodological approach across the two countries, Rwanda, and SA. This protocol was slightly adapted by each country’s research team for local implementation. Additionally, some aspects of the methodology changed during the data collection phase; for instance, in SA, all stages of data collection presented were conducted online, due to COVID-19 public health restrictions.

#### Desk review

We conducted a desk review of policy documents to identify population-level policies, programs and supporting environment interventions (henceforth referred to simply as ‘interventions’) targeting risk factors for diabetes and hypertension currently implemented in Rwanda and SA. The respective country teams each conducted online searches on various government and non-government websites such as WHO and databases as described [23] for relevant documents describing population level interventions. We also consulted key ministry of health officials to recommend relevant documents and websites relevant for the desk review. A total number of 62 documents were reviewed (18 documents from Rwanda and 44 documents from SA). The desk review was carried out from February-September 2019 in Rwanda and from August-November 2019 in SA. A standardized excel template for data extraction was used to document relevant information relating to what interventions have been implemented including the coverage, target audience and any information regarding impact evaluations if any.

#### Stakeholder mapping

Then a stakeholder mapping was done from national to provincial levels to describe the landscape of key players in the country working on the prevention of diabetes and hypertension. This was an essential step to identify and subsequently contact those individuals critical for designing, implementing, maintaining and evaluating population-level interventions of interest. The mapping of stakeholders started with reaching out to relevant personnel at the Ministry of Health and other governmental or non-governmental agencies known to be actively involved in the implementation of population-level interventions, and then used snowballing methods to identify and map further relevant stakeholders.

The identified key stakeholders included the national department of health, provincial departments of health, agriculture, sports, universities and research institutions (e.g. Stellenbosch University, University of Cape Town, SA Medical Research Council), non-governmental organization (e.g. the NCD alliance in Rwanda), the Heart Foundation, various industries (in SA) and regulatory agencies. A stakeholder mapping matrix tool [24] was used for the mapping.

#### Survey among key stakeholders

We subsequently conducted a survey with 60 and 12 key stakeholders in Rwanda and SA, respectively, with the aim of identifying further relevant population-level interventions and of understanding more about these, including information on target population, coverage, and existing process or impact evaluations. We selected participants for the survey from the stakeholder mapping based on their expertise and likelihood of being informed about the topic. The selected stakeholders were then contacted by phone to confirm their eligibility in terms of current employment, area of expertise and willingness to partake in the survey. The survey was conducted face to face from June-August 2019 in Rwanda and online from March-May 2020 in SA. The contents of the data collected from the survey was purely narrative (names of the interventions, coverage, target population, evaluation if any, etc) and could not be analyzed quantitatively using descriptive or analytical statistics. The data from this step was categorized into thematic areas and triangulated with the findings of the desk review in order to have a more comprehensive picture of implemented interventions in both countries.

#### Key informant interviews

We undertook key informants’ interviews (KIIs) to explore barriers/gaps and facilitators/opportunities for effective implementation of population level interventions in the respective countries. Participants for the KIIs were drawn from those stakeholders having participated in the surveys. The selection was guided by the participants’ experience and seniority in implementing population level interventions. For example, we targeted individuals whose focus is NCDs, heads of NCD units (from both private and public institutions) and members of national NCD technical working group. Ten KIIs took place face-to-face from August-September 2019 in Rwanda. In SA, 13 KIIs were conducted from April-June 2020. Qualitative data from interviews were thematically analysed using Atlas ti. (Rwanda) and Nvivo (SA), which provided a detailed description of challenges, weaknesses, gaps and opportunities in terms of advancing population-level interventions targeting risks factors for hypertension and diabetes. The findings from the KIIs are described elsewhere [22, 25].

#### Consultative workshops

After producing a preliminary analysis of the findings from the desk review, the survey and the qualitative interviews, which focused on the strengths, weaknesses, opportunities and threats to the successful implementation of population-level interventions targeting risk factors for diabetes and hypertension, we conducted a one-day consultative workshop with key NCD stakeholders in each of the two countries. While in Rwanda the workshop was held in-person with 34 stakeholders in November 2019 before the Covid-19 pandemic, in SA, the workshop was conducted online with 10 participants in December 2020 during the Covid-19 pandemic. These workshops sought to discuss the findings of the preliminary analysis and to consolidate gaps as well as opportunities for advancing population-level interventions. Workshop participants were asked to comment on the findings and to suggest new insights or recommendations, which could make the findings and interpretations of the situational analysis clearer, more comprehensive and/or more appropriate. Workshop participants were drawn from survey participants and KII participants, selected according to their expertise in implementing population level interventions of interest. After compiling and integrating the additional information, a comprehensive situational analysis report was finalized.

### Challenges encountered and lessons learnt

As illustrated in Fig. 1 and described in detail above, the situational analysis involved multiple stages of quantitative and qualitative data collection and analysis. This involved the planning and conduct of multiple stages of research, the design of multiple data collection instruments, and the engagement of participants over time – throughout this process we experienced multiple challenges, but also learned multiple lessons. After completing the situational analysis, the authors held multiple team meetings over the course of two months to identify, reflect on and discuss challenges and lessons learned in the process of employing the methodology. Through these reflective meetings, we identified three aspects in the planning and conduct of the multi-stage mixed methods situational analysis, which we felt were most influential and relevant both regarding challenges encountered and lessons learned: i) stakeholder recruitment, engagement and retainment; ii) collection, utilization and triangulation of multiple sources of data; and iii) evolving circumstances, particularly related to the Covid-19 pandemic.

#### Recruiting, engaging and retaining key stakeholders

Several challenges in the planning and conduct of the situational analysis related to the recruitment, engagement and retainment of key stakeholders were encountered. With regards to recruitment of key stakeholders, especially the non-health sector, who do not consider themselves as making/influencing policies, engagement in a multi-stage research design was quite challenging, given that they did not perceive themselves as key players in making/ influencing policies. This led to their reluctance and unresponsiveness to online correspondence until the face-to-face visit was made to explain their participation relevance in person. We noticed a particular unwillingness of private sector, especially industries, to participate in the study. This is notable, as their production of essential products such salt, oil and sugar, makes them important potential stakeholders in implementing population level intervention targeting risk factors for diabetes and hypertension. Additionally, due to the sequentially conducted mixed-methods nature of the situational analysis, the stakeholders were invited to participate in different stages of the study. However, this meant that these stakeholders had to be engaged in the study for a longer period, making the retainment of these stakeholders through the end of the study quite challenging due to prioritization of usual work demands over the research demands.

As lessons learnt, there is a need to work with stakeholders to develop strategies that will keep stakeholders engaged in research that might involve multiple stages of data collection. Early, more intensive and targeted involvement of stakeholders could be beneficial to help ensure buy-in and longer-term engagement is crucial in this kind of multi-stage study. Relevancy to stakeholders outside of the health sector should be consciously considered to keep their continuous engagement in the study. Researchers should learn how our research processes and formats can be improved and communicated so that stakeholders perceive their commitment and continued involvement in the research as a valuable use of their time as well as a benefit to public health. This aspect may be the strongest for stakeholders outside of the health sector, for whom a health-related study may not always be considered a priority, particularly for those in the industry, where there may be concerns about economic loss.

#### Collection, utilization and triangulation of multiple sources of data

Further challenges we encountered related specifically to the collection, utilization and triangulation of multiple sources of data collected over a prolonged period. Country-specific limitations existed for specific sources of data. For example, in Rwanda, the desk review identified only a very limited number of informative documents on population-level interventions, due to the lack of such documents publicly accessible. Due to this limitation, the mapping of the relevant stakeholders took longer than expected.

An important challenge in such a multi-stage study is how time-consuming it is for both researchers and stakeholders. Same participants who participated in the survey also took part in both KIIs and consultative workshops.

This approach was sometimes perceived by participants as redundant; this represents a challengeparticularly for practitioners and policy makers who had other important demands, such as of Covid-19 in SA.

Through the collection, utilization and triangulation of multiple sources of data over time, we learnt the importance of balancing different methodological steps to reinforce and complement one another, without being duplicating information across steps. Triangulation of different sources of data was useful to address the objectives of the situational analysis. The different steps targeted and yielded unique pieces of data, concepts and perspectives which complemented one another. Thus, in weaving these various strands together, we were better able to understand the full picture on the status of the implementation of population level interventions targeting risk factors of diabetes and hypertension and the progress made in implementing the WHO Best buys interventions for NCDs, as well as contextual challenges related to policy formulation, adoption and implementation processes. The initial quantitative survey allowed us to identify population-level interventions targeting risk factors for diabetes and hypertension (and a lack of interventions) that we could further explore through the qualitative KIIs. These KIIs further helped to explore, with those institutions and individuals responsible for such interventions, what barriers and facilitators to their design, implementation, evaluation and sustainment are. The consultative workshops with key stakeholders as a final stage were critical in making sure that our findings were appropriate, understandable, and informative. The participative involvement of stakeholders across stages fostered inter-sectoral engagement, dialogue and allowed us to contextualize the results and ensure that the implications for research and policy were correctly interpreted and presented and did justice to the multiple perspectives and local contexts.

#### Evolving circumstances

Finally, evolving circumstances related to the study but also to the external context represented a challenge throughout the conduct of the situational analysis. In SA particularly, much of the study period coincided with the Covid-19 pandemic, which created a series of challenging circumstances. Shifting of priorities of stakeholders contributed to poor response specifically for the survey. The data collection activities were conducted at the onset of the Covid-19 pandemic which heavily weighted on and stressed the health sector and their stakeholders. This has led to reluctance/ non-availability of the participants due to the busy schedules. In addition, the lockdown and other hygiene measures meant the inability to continue with in-person data collection, as specified a priori in the study protocol. In SA, as a result, both the survey and the KIIs were conducted online as an adjustment to lockdown restrictions.

From these challenges, we learned that in the future, we need to work with stakeholders to learn how our research processes and formats can be improved and communicated so that they perceive their commitment and continued involvement in the research as a valuable use of their time as well as a benefit to public health. In addition, non-health stakeholders such as industry involved in food and beverages require a new strategy of engagement given the contentious issues related to public health gains versus economic gains. In Rwanda, those stakeholders who were embedded in the CEBHA+ integrated knowledge translation strategy, thus who were involved longer-term in the overarching project, tended to engage more readily and actively with the situational analysis. In SA, identifying a champion or focal person for the engagement in their respective workplace was helpful to promote regular engagement. This could suggest that early, more intensive, and targeted involvement of stakeholders could be beneficial to help ensure buy-in and longer-term engagement. Flexible, yet appropriate adaptations to methodology in SA ensured that challenges could be met along the way and that the original study aims could be accomplished without sacrificing study quality.

## Discussion and conclusions

### Summary of methodology and lessons learnt

We conducted a multi-stage mixed-methods situational analysis of population-level interventions targeting risk factors of diabetes and hypertension in Rwanda and SA. This paper describes and reflects upon the main challenges and lessons learnt in that process. We identified three aspects in the planning and conduct of the situational analysis that capture multiple challenges encountered and lessons learnt i) stakeholder recruitment, engagement and retainment; ii) collection, utilization and triangulation of multiple sources of data; and iii) evolving circumstances, particularly related to the Covid-19 pandemic. The reflections highlight that recruiting, engaging and retaining stakeholders over time proved challenging. This could be due to busy schedules of targeted stakeholders and competing interests. However, we also deemed that their involvement was critical to ensure that the findings were well-informed and appropriate. We also found the collection utilization and triangulation of multiple sources of data over time and across two countries challenging. However, the different steps targeted and yielded unique pieces of data, concepts and perspectives which complemented one another. Thus, in weaving these various strands together, we were better able to understand the full picture on the status of the implementation of population-level interventions targeting risk factors of diabetes and hypertension, as well as contextual challenges. Evolving circumstances due to the Covid-19 pandemic challenged the conduct and completion of the study. We learnt, however, that flexible adaptations to other forms of data collection allowed these challenges to be addressed and for original study aims was esteemed to be met.

### Conclusions and recommendations for further research

Situational analysis in the context of health interventions is an important, yet underutilized tool for identifying and understanding existing interventions, as well as the multiple interacting factors, including context, linked to these interventions [15, 16, 21]. Such information is critical for policy evaluation and advancement, especially in low-resource settings. This experience from conducting this situational analysis using multi-stage mixed methods approach can be used by researchers planning to explore the use of health policies at the population-level.

We encourage others wishing to conduct situational analysis to build on and refine our methods and to consider our lessons learnt when planning and conducting their situational analysis. We believe that the methods used, challenges encountered as well as the lessons learnt are common, especially in similar settings (LMICs countries) and would be informative for other researchers conducting a similar study. Finally, while the use of mixed methods research can be resource intensive, they should be considered when conducting situational analyses to ensure a more robust and comprehensive analysis of global health policy and health systems.

## Data Availability

The datasets used and/or analysed during the current study available from the corresponding author on reasonable request.

## Abbreviations

BMBF: German Federal Ministry of Education and Research
CEBHA+: Collaboration for Evidence-based Healthcare and Public Health in Africa
CVDs: Cardiovascular Diseases
KIIs: Key informant interviews
LMU: Ludwig Maximilian University of Munich
LMICs: Low and middle-income countries
NCDs: Non-Communicable Diseases
IBE: Institute of Biometry and Epidemiology
SA: South Africa
SADHS: South African Demographic and Health Survey
STEPS: WHO STEPwise approach to NCD risk factor surveillance
SSA: Sub-Saharan Africa
SWOT: Strengths, Weaknesses, Opportunities, and Threats
WHO: World Health Organization

## References

[1] World health Organization (2021). Non-communicable diseases.

[2] World Health Organization (2021). Cardiovascular diseases (CVDs) Factsheets.

[3] Mendis S, Graham I, Narula J (2022). Addressing the Global Burden of Cardiovascular Diseases; Need for Scalable and Sustainable Frameworks. Glob Heart..

[4] World Health Organization (2019). Fact sheets-Hypertension.

[5] Cho NH, Shaw JE, Karuranga S, Huang Y, da Rocha Fernandes JD, Ohlrogge AW (2018). IDF Diabetes Atlas: Global estimates of diabetes prevalence for 2017 and projections for 2045. Diabetes Res Clin Pract.

[6] World Health Organization (2021). Non-communicable diseases.

[7] Charlotte Munganyinka Bavuma, Jean Berchmans Niyibizi, Leopold Bitunguhari, Ruth McQuillan, Sarah Wild. Prevalence and characteristics associated with diabetes mellitus and impaired fasting glucose among people aged 15 to 64 years in rural and urban Rwanda: secondary data analysis of World Health Organization surveillance data. PanAfrican Med J. 2022 [cited 2022 May 30];41(115). Available from: https://www.ncbi.nlm.nih.gov/pmc/articles/PMC8994463/pdf/PAMJ-41-115.pdf. 10.11604/pamj.2022.41.115.30682 PMC8994463 35465373

[8] National Department of Health (NDoH), Statistics South Africa (Stats SA), South African Medical Research Council (SAMRC) and I 2019. South Africa Demographic and Health Survey 2016 [Internet]. Pretoria, South Africa, and Rockville, Maryland, USA; 2016. Available from: https://dhsprogram.com/pubs/pdf/FR337/FR337.pdf.

[9] World Health Assembly. Follow-up to the Political Declaration of the High-level Meeting of the General Assembly on the Prevention and Control of Non-communicable Diseases. 2013 [cited 2019 May 3]. 1–55. Available from: https://apps.who.int/gb/ebwha/pdf_files/WHA66/A66_R10-en.pdf.

[10] NDOH, Pretoria SA (2013). National Department of Health: Strategic Plan for the Prevention and Control of Non-communicable Diseases 2013–2017.

[11] World Health organization (2017). PREVENTING NONCOMMUNICABLE DISEASES (NCDs) BY REDUCING ENVIRONMENTAL RISK FACTORS.

[12] WHO (2017). “Best buys” and other recommended interventions for the prevention and control of noncommunicable diseases. World Heal Organ.

[13] WHO. Global action plan for the prevention and control of noncommunicable diseases 2013-2020. World Heal Organ. 2013 [cited 2022 Sep 20];102. Available from: http://apps.who.int/iris/bitstream/10665/94384/1/9789241506236_eng.pdf.

[14] Tejeda MJ. Book Review: Clarke, A. E. (2005). Situational analysis: Grounded theory after the postmodern turn. Thousand Oaks, CA: Sage. 2016 [cited 2022 Sep 20];10(2):390–2. Available from: https://journals.sagepub.com/doi/10.1177/1094428106290198.

[15] Murphy JK, Michalak EE, Colquhoun H, Woo C, Ng CH, Parikh SV (2019). Methodological approaches to situational analysis in global mental health: a scoping review. Glob Ment Heal..

[16] Martin W, Pauly B, MacDonald M (2016). Situational Analysis for Complex Systems: Methodological Development in Public Health Research. AIMS public Heal..

[17] Singh A, Owusu-Dabo E, Dobbie F, Mdege N, McNeill A, Britton J (2020). A situational analysis of tobacco control in Ghana: progress, opportunities and challenges. J Glob Heal reports..

[18] Mokitimi S, Schneider M, de Vries PJ (2022). A situational analysis of child and adolescent mental health services and systems in the Western Cape Province of South Africa. Child Adolesc Psychiatry Ment Health..

[19] Makau-Barasa LK, Manirakiza A, Carvalho AL, Rebbeck TR. Prostate Cancer Screening, Diagnostic, Treatment Procedures and Costs in Sub-Saharan Africa: A Situational Analysis. Cancer Control. 2022 [cited 2023 Jan 19];29. Available from: /pmc/articles/PMC8973068/. 10.1177/10732748221084932 PMC8973068 35350915

[20] Monedero-Recuero I, Gegia M, Wares DF, Chadha SS, Mirzayev F (2021). Situational analysis of 10 countries with a high burden of drug-resistant tuberculosis 2 years post-UNHLM declaration: progress and setbacks in a changing landscape. Int J Infect Dis..

[21] Bentley ME, Johnson SL, Wasser H, Creed-Kanashiro H, Shroff M, Fernandez-Rao S (2014). Formative research methods for designing culturally appropriate, integrated child nutrition and development interventions: An overview NIH Public Access. Ann N Y Acad Sci..

[22] Nganabashaka JP, Ntawuyirushintege S, Niyibizi JB, Umwali G, Bavuma CM, Byiringiro JC (2022). Population-Level Interventions Targeting Risk Factors for Hypertension and Diabetes in Rwanda: A Situational Analysis. Front Public Heal..

[23] Uwimana-Nicol J, Hendricks L, Young T (2021). Population-level interventions targeting risk factors of diabetes and hypertension in South Africa: a document review. BMC Public Health..

[24] Astrea Balane M, Palafox B, Palileo-Villanueva LM, Mckee M, Balabanova D (2020). Enhancing the use of stakeholder analysis for policy implementation research: towards a novel framing and operationalised measures. BMJ Glob Heal..

[25] Nicol JU, Hendricks L, Young T. Challenges and enablers for implementation of WHO â€˜Best buysâ€^TM^ interventions targeting risk factors of diabetes and hypertension in South Africa: a mixed methods study. PAMJ 2022; 43:215 [Internet]. 2022 Dec 30 [cited 2023 Jan 19];43(215). Available from: https://www.panafrican-med-journal.com/content/article/43/215/full. 10.11604/pamj.2022.43.215.31547 PMC10038766 36974315

